# Small Supernumerary Marker Chromosomes in Humans: From Classical Cytogenetics to High-Resolution Cytogenomics

**DOI:** 10.3390/ijms27219812

**Published:** 2026-11-01

**Authors:** Nikolay B. Rubtsov, Tatyana V. Karamysheva, Anna A. Kashevarova, Igor N. Lebedev

**Affiliations:** 1 Research Institute of Medical Genetics, Tomsk National Research Medical Center of the Russian Academy of Sciences, 634050 Tomsk, Russia; kary@bionet.nsc.ru (T.V.K.); anna.kashevarova@medgenetics.ru (A.A.K.); igor.lebedev@medgenetics.ru (I.N.L.); 2 Institute of Cytology and Genetics of the Siberian Branch of the Russian Academy of Sciences, 630090 Novosibirsk, Russia

**Keywords:** small supernumerary marker chromosome (sSMC), cytogenomic diagnostics, metaphase chromosome microdissection, array-based comparative genomic hybridization, long-read genome sequencing, optical genome mapping, sSMC pathogenicity

## Abstract

Small supernumerary marker chromosomes (sSMCs) are additional chromosomal elements smaller than or equal to chromosome 20, with a prevalence of ~0.044% in the population. Their clinical impact ranges from asymptomatic carriage to severe congenital, neurodevelopmental, and other disorders. Accurate diagnosis and interpretation remain challenging due to their small physical size, frequent tissue-specific mosaicism, and variable expression of similar gene content across different genetic backgrounds, which is well known for numerous clinically significant gene variants. This review synthesizes current knowledge on sSMC biology, diagnostics, and clinical management, highlighting the paradigm shift from classical cytogenetics to high-resolution genomic and cytogenomic approaches, including long-read genome sequencing combined with telomere-to-telomere chromosome assemblies. We present an updated sSMC classification based on sSMC DNA content and structural organization, discuss molecular mechanisms of sSMC biogenesis, and critically evaluate the diagnostic utility of microarray comparative genomic hybridization, whole-genome sequencing, and chromosome microdissection-based approaches. We also discuss breakpoint positions in sSMC formation, sSMC localization in nuclear spatial compartments, and tissue distribution patterns as factors with possible influence on the sSMC manifestation. Some of these properties might be used as criteria for assessing the pathogenic potential of sSMCs, while others require further investigation. Furthermore, we refine pathogenicity assessment frameworks by incorporating sSMC content and morphology. By integrating cutting-edge molecular cytogenetics with standardized interpretive guidelines, this narrative review provides a useful framework for precise sSMC characterization, which is essential for genetic counseling and targeted clinical management.

## 1. Introduction

Human small supernumerary marker chromosomes (sSMCs) are structurally diverse extrachromosomal elements in karyotype that arise either *de novo* or through inheritance. Numerous studies have focused on identifying and describing the morphology, inheritance patterns, structural organization, DNA content, and telomere-to-telemore (T2T) assembly of sSMCs. In this narrative review, we analyze the results presented in articles published in peer-reviewed medical and biological journals included in the medical databases: Global Index Medicus (World Health Organization), Web of Science (https://www.webofscience.com), Embase (www.embase.com), PubMed (https://pubmed.ncbi.nlm.nih.gov/), and the specialized ChromosOmics: Small Supernumerary Marker Chromosomes Database (https://cs-tl.de/DB/CA/sSMC/0-Start.html, accessed on 17 September 2026) [1]. The reviewed data encompass information from the first description of sSMC in 1961 to the T2T sSMC assemblies generated in 2026. It should be noted that, despite the vast number of publications dedicated to sSMC diagnostics and the description of their specific variants, the use of diverse methods—including those employed at different points in time—the limited sampling of tissues, and the inability to validate proposed concepts and mechanisms make it significantly difficult to construct a unified understanding of how sSMCs arise, how diagnostic methods for them have evolved, and how their potential pathogenicity might be assessed.

Recent articles and preprints from bioRxiv.org were also screened; however, only peer-reviewed publications were ultimately included in the final analysis. The literature search and data analysis focused on case reports, sSMC detection methods, mosaicism analysis, and data on the structural and molecular organization of sSMCs, their inheritance and effects on the carrier phenotype.

By cytogenetic convention, sSMCs are defined as small extra chromosomes equal to or smaller than chromosome 20 in the same metaphase spread that cannot be unequivocally identified using conventional chromosome banding alone [1]. Notable exceptions to this rule are inverted duplications of the short arms of five submetacentics and chromosome 20 [i(5)(p10), i(8)(p10), i(9)(p10), i(12)(p10), i(18)(p10), and i(20)(p10)], which can be identified by GTG- or DAPI-banding but are traditionally classified as sSMCs [2]. With an estimated prevalence of ~0.044% in the general population, sSMCs pose a significant diagnostic challenge in both prenatal diagnostics and postnatal clinical genetics. Although approximately 70% of carriers are phenotypically normal, the remaining 30% exhibit a broad spectrum of clinical manifestations, including intellectual disability, global developmental delay, epilepsy, congenital malformations, reproductive dysfunction, and high fetal and postnatal mortality [1,2,3,4,5].

The clinical manifestations of an sSMC are governed by its gene content and gene dosage effects, structural topology, tissue distribution, parental origin, epigenetic modifications and probably its spatial positioning within the interphase nucleus. The first sSMC was identified in 1961 using routine metaphase chromosome staining [6]. Historically, sSMC detection relied on metaphase chromosome staining. Later, its origin and content were determined using fluorescence *in situ* hybridization (FISH) and new cytogenomic techniques [7]. The possibilities of the molecular cytogenetic techniques based on metaphase chromosome preparations are limited by the resolution of metaphase or prometaphase chromosome analysis and the availability of DNA probes. The integration of high-resolution microarray platforms [8], long-read genome sequencing (lrGS) [7,9], optical genome mapping (OGM) [10,11], complete telomere-to-telomere genome assemblies [12], and metaphase chromosome microdissection [13] has fundamentally transformed sSMC diagnostics (Table 1).

lrGS, combined with complete telomere-to-telomere sSMC assemblies, now enables base-pair resolution of breakpoint junctions (BPJs), and the precise reconstruction of structural complexity [7]. Nevertheless, sSMC remains a significant challenge for many cytogenetic laboratories due to technical and infrastructural limitations and the absence of standardized interpretation frameworks.

In this review, we synthesize current knowledge on sSMC biology and cytogenetic diagnostics, present an updated classification system, discuss the molecular mechanisms driving sSMC biogenesis, and critically evaluate contemporary genomic technologies. We also propose a refined pathogenicity assessment framework that integrates sSMC molecular and architecture composition, tissue-specific mosaicism to improve prognostic accuracy, genetic counseling, and evidence-based clinical decision-making. Some of the discussed sSMC characteristics can be used—and are already being used—as criteria for assessing the pathogenic potential of sSMCs, while others, such as the compartmentalization and spatial positioning of sSMCs in the interphase nucleus, remain hypothetical and require experimental confirmation.

The review includes a discussion of the data on sSMCs published from the time of the first sSMC description through September 2026 and the techniques applicable to sSMC diagnostics. Notably, greater attention has been paid to the detailed characterization of sSMCs. This approach has allowed a deeper investigation into the mechanisms of their potential origin and their clinical manifestations. However, it may also have introduced a potential bias regarding the cases included in the review.

## 2. Challenges in Assessing sSMC Pathogenicity

In addition to DNA content, several biological and technical factors complicate the accurate assessment of the sSMC clinical impact. They could encompass tissue-specific mosaicism (including the heterogeneity of sSMC copies), epigenetic chromatin modifications, such as X-inactivation for sSMC derived from chromosome X, structural complexity, possible variable spatial positioning within the interphase nucleus, secondary alterations of chromatin topology in other chromosomes, and variable manifestation of the same inherited sSMC across different individuals due to the diversity of personal genomes. The ChromosOmics Database, created and supported by Thomas Liehr and his colleagues [1], remains the most comprehensive resource for sSMC phenotyping. Unfortunately, many entries in the ChromosOmics Database are based on data obtained several years ago using outdated cytogenetic techniques, and the influence of individual genomic background on sSMC manifestation remains largely unaddressed. To resolve this problem, large cohorts with fully assembled individual genomes and high-resolution sSMC characterization are required. Current studies also face numerous practical limitations. Available biopsy material is typically restricted to peripheral blood, umbilical cord blood, amniocytes, and skin fibroblasts, leaving conclusions about systemic mosaicism incomplete. The accuracy of FISH-based DNA probe localization on metaphase and prophase chromosomes is limited by the resolution of chromosomal bands/subbands, as well as the availability and composition of the DNA probes used. Bulk analysis techniques, such as array Comparative Genome Hybridization (aCGH) and Whole-Genome Sequencing (WGS), rely primarily on analyzing DNA isolated from thousands of cells, which precludes the analysis of DNA from individual sSMC copies. However, individual sSMC copies may be analyzed by sequencing of microdissected DNA libraries generated from individual sSMC copies [28]. Today, sSMC diagnostics is mainly focused on the analysis of sSMC DNA content, whereas structural, spatial and epigenetic profiling requires the application of additional methodologies not yet routine in clinical diagnostics. It should also be considered that an optimal and efficient strategy of sSMC diagnostics depends on the type of analyzed sSMC, highlighting the importance of the selection of relevant techniques. For instance, the application of aCGH and standard WGS is complicated for cases with low-level mosaicism and inefficient for the analysis of purely heterochromatic sSMC [29]. The distinct capabilities and limitations of current sSMC diagnostic methods necessitate a comprehensive approach that integrates various molecular cytogenetic and cytogenomic technologies.

## 3. sSMC Mosaicism

Differential distribution of sSMCs across tissues can drastically modify their phenotypic manifestation [30]. Unfortunately, current studies on sSMC mosaicism are largely restricted to a few accessible tissues. While low sSMC frequency in peripheral blood is often assumed to reflect low sSMC frequency elsewhere, this is not universally true. For example, the i(12)(p10) marker chromosome associated with Pallister–Killian syndrome (PKS) is typically rare in the cells of peripheral blood but enriched in skin fibroblasts [31]. The low frequencies of i(12)(p10) in peripheral blood could be associated with tissue-specific selection pressure. It is worth noting that *in vitro* cell culturing prior to karyotyping can also distort sSMC frequency estimation due to clonal selection. It is also important to note that the mosaicism pattern of an inherited sSMC in a child may differ from that observed in the parent. Although the absence of abnormalities in the parent is often considered evidence of a benign sSMC nature, it does not guarantee a benign manifestation in the offspring. Such phenotypic variability can arise from differences in sSMC distribution across various tissues. When assessing the likelihood of mosaicism concordance between parents and their offspring, the following points should be considered: sSMCs arising from biarmed chromosomes are more prone to form mosaic variants than those derived from acrocentrics [30], and in some cases, such as the i(12)(p10) marker chromosome, sSMC distribution across tissues is probably shaped by strong selective pressure at the cellular or organismal level [31].

## 4. sSMCs with Different DNA Content

In general, sSMCs can derive from any chromosome. They show significant diversity in their DNA content, including the size of their euchromatic regions (ECRs) and their gene content [1]. Most sSMCs contain an active centromere and pericentromeric region derived from the original chromosome; however, in rare cases, a neocentromere could be present instead of an original one [32]. Some sSMCs can additionally contain ECRs of different origins [7]. Most sSMCs contain telomeric repeat clusters, whereas some lack them [7,33]. Telomeres may or may not be detected on a ring sSMC (rsSMC) and the absence of telomeres does not automatically guarantee that the analyzed sSMC is ring-shaped [33]. Confirmation of the rsSMC structure requires additional studies [7,28].

The detection of an sSMC immediately raises two critical questions: Is it *de novo* or inherited? Does it contain ECRs, or is it entirely heterochromatic? A *de novo* sSMC is associated with an additional risk of uniparental disomy (UPD) for the homologous chromosome, which may contain known imprinted genes, potentially requiring targeted UPD testing in cases involving an sSMC derived from chromosomes 6, 7, 8, 11, 14, 15, or 20 [34]. Although an ECR-negative sSMC lacks genes and generally does not lead to genetic imbalance, a *de novo* sSMC could indicate an increased risk of other chromosomal rearrangements that might accompany sSMC formation. The mechanisms of UPD formation associated with *de novo* sSMCs have been previously discussed [35,36,37]. Data exist on repeat transcription in C-positive chromosome regions [38], but heterochromatic variations usually do not show clinical manifestations. The size of the C-positive regions of chromosomes 1, 9, 16, and the Y varies within a wide range. Furthermore, individuals with a reduced number of centromeres per cell resulting from Robertsonian translocations of acrocentric chromosomes, as well as those with an additional Y chromosome, exhibit normal or near-normal phenotypes [39]. Furthermore, numerous studies have showed that sSMCs composed solely of centromeres, pericentromeric heterochromatin, and telomeric repeats do not disrupt normal development [1,40].

In contrast to heterochromatic sSMC, sSMCs containing an ECR introduce partial aneuploidy and require detailed molecular characterization of their DNA content. Their clinical manifestations range from benign to highly pathogenic. The pathogenicity of an sSMC is expected to depend on its gene content, ECR size, structural organization, epigenetic modifications, and several other factors. Notably, the level of sSMC mosaicism plays a key role in determining pathogenicity, significantly mitigating it if the sSMC is present at a low frequency in cells of the affected tissues.

The size of an sSMC ECR varies dramatically, ranging from very small to comparable with a small human chromosome [1]. The marker chromosomes i(5)(p10), i(8)(p10), i(9)(p10), i(12)(p10), i(18)(p10), and i(20)(p10) are inverted duplications of the short arms of the involved chromosomes. Although their identification is possible via GTG- or DAPI-banding, their confirmation using whole-chromosome paints (WCPs) [41,42] or arm-specific chromosome paints (ACPs) [43], as well as aCGH, is strongly recommended [44]. The application of aCGH and WGS is useful for evaluating the copy number of ECRs within the sSMC. Unfortunately, these techniques cannot distinguish between a duplicated region located on the sSMC and a segmental duplication on the intact linear chromosome. Furthermore, sSMCs consisting of a single arm of the original chromosome in all cells could not be distinguished from inverted duplications of the short arm present only in half of the cells. aCGH shows low efficiency in the case of low sSMC frequency. Both aCGH and WGS cannot reveal an sSMC containing no ECR. Thus, these techniques should be applied in combination with routine or molecular cytogenetics.

sSMCs consisting of the short arm of submetacentric chromosomes or chromosome 20 contain large ECRs often exhibit an intact structure of the entire chromosome arm, leading to classical trisomy or tetrasomy of the corresponding arm. Key diagnostic considerations for these sSMCs include detecting interstitial rearrangements, evaluating the ECR copy number, and assessing mosaicism. A single copy of the involved chromosome arm combined with a lower sSMC frequency in the cells of certain tissues could result in milder phenotypic effects [45]. While the Pallister–Killian syndrome (PKS) is associated with an i(12)(p10) marker that typically shows a low frequency in peripheral blood cells and a higher frequency in fibroblasts, the classical phenotype of PKS was observed in a patient carrying an sSMC consisting of a single copy of 12p in 50% of peripheral blood cells [44]. It should be noted that aCGH revealed an additional interstitial duplication of the 12p12.1–p13.1 region, which affects the genes critical for PKS and might explain the observed phenotype. It was found that, besides the dosage effect of additional gene copies, trisomy of the whole chromosome could change the spatial organization of the interphase nucleus, the structure of topologically associated domains (TADs) in other chromosomes, and the transcription of the genes located in other chromosomes [46]. A similar impact of the sSMCs on genome spatial organization and gene transcription has not been studied yet. Nevertheless, given the complex genotype–phenotype correlations of sSMCs, such a hypothesis cannot be ruled out.

sSMCs derived from chromosomes 15 and 22 are distinctive, being the two most common sSMCs in the general population [47]. The high frequency of chromosome rearrangements in proximal regions of q arms of these chromosomes is driven by low-copy repeats and inversions located in these regions [48,49,50]. At the 15q11.2 → q13 region, five breakpoint hotspots (BPs) are well known: BP1 and BP2 at 15q11.2, BP3 at 15q13.1, BP4 at 15q13.2, and BP5 at 15q13.3 [51]. Additionally, BP6 was revealed at 15q14 [52]. It should be noted also that the 15q11 → q14 region is characterized by segmental duplications and inversions of different sizes, making the region very unstable.

Rearrangements can involve DNA breakage and rejoining within a single chromatid, or they may involve DNA from a homologous chromosome. Low-copy repeats (LCRs) and the regions located between them could be present in inverted orientations on homologs. Recombination occurring within the inverted region yields the classic products of crossing-over associated with paracentric inversion: a dicentric isochromosome and an acentric fragment. As a result, the sSMCs derived from chromosomes 15 and 22 are mostly inverted duplications of the region that includes the short arm and proximal region of the long arm of the original chromosome. They were named isodicentric chromosomes (idic(15), idic(22)) or inv dup(15) and inv dup(22). In fact, these sSMCs contain two centromeric regions but only one active centromere [53] and some of them are not the typical inverted duplications.

Most idic(15) chromosomes result from breakage–fusion events at BP3 (BP3:BP3), accounting for 61% of cases, and give rise to symmetric isodicentric chromosomes [49]. The next most frequent variant of idic(15) is an asymmetric dicentric chromosome derived from a rearrangement involving BP3 and BP5 (29%) [49]. Other variants are minor: BP5:BP5 (~4%), BP2:BP3 (~3%), BP2:BP2 (~1%), BP3:BP4 (~1%), and BP4:BP4 (~0.6%) [49]. As with other sSMCs, the manifestation of sSMC(15) depends on its ECR and mosaicism pattern. In cells containing idic(15) formed by BP3::BP3, two additional copies of the 15pter-q13.1 region lead to partial tetrasomy on chromosome 15 and Dup15q syndrome (hypotonia, developmental delay ranging from mild to severe, behavioral abnormalities, sleep disturbances, and epilepsy) [54,55,56]. This is the most common variant. The second one is sSMC(15) formed with breakage–fusion events at BP3::BP5. In addition to the BP3::BP3 variant, it leads to trisomy on the region located between BP3 and BP5, which results in a more severe Dup15q syndrome manifestation [49]. A similar Dup15q syndrome manifestation was observed for the BP3::BP4 variant of sSMC(15). Carriers of sSMC(15) containing a very small ECR, such as the one derived from BP2:BP2 rearrangement (~2 Mb), show a normal or near-normal phenotype.

However, the karyotype of 20–25% of patients with Dup15q syndrome contains no sSMC. An additional copy of the 15q11.2-q13 region is located within chromosome 15. Interstitial duplication of 15q11.2–q13.1, in contrast to most sSMC(15)s, leads to partial trisomy of this region, the size of which can vary [49]. Differences in the manifestation of the additional interstitial copy of the 15q11.2-q13 region and additional copies located in sSMC(15)s were identified. Although some phenotypic features of inv dup(15) carriers with tetrasomy for the BP1–BP3 region and of subjects with interstitial duplication are similar; patients with interstitial duplication show a more subtle phenotype. While the clinical manifestation of inv dup(15) (developmental delay/intellectual disability, autism, central hypotonia, minor dysmorphisms, and seizures) ranges mostly from mild to profound, in 13 of 14 patients with interstitial duplication only mild to moderate developmental delay was revealed [57].

It should be noted that, besides its highly complex organization and gene regulation, the 15q11–q13 region is subject to imprinting. Gene expression in this region differs between maternal and paternal chromosomes. The manifestation of 15q11.2–q13.1 interstitial duplication when inherited from the mother or the father also differs. Maternal interstitial duplication of 15q11.2–q13.1 is mostly associated with neurodevelopmental and physical disorders, while patients with paternal interstitial duplication usually show normal development or very mild symptoms [57]. 

The specific clinical manifestations of inv dup(15) should be taken into account. sSMC(15)s arising from breakpoints at BP1 or BP2 containing no ECR or a very small ECR lead to no or only a very small genetic imbalance. The 15q11.2 BP1–BP2 interval contains four highly conserved, nonimprinted genes: *NIPA1*, *NIPA2*, *CYFIP1*, and *TUBGCP5* [58]. The additional copies of such sSMCs(15) show no evident pathogenic effect. However, *de novo* emergence of such sSMCs(15) could be accompanied by abnormal development through the formation of UPD(15), leading to Prader–Willi syndrome (PWS) or Angelman syndrome (AS) [59,60]. *De novo* sSMC emergence may be accompanied by UPD in 5–10% of all *de novo* sSMC cases. Partial trisomy rescue occurring during early embryonic development is considered to be the main mechanism of such UPD formation [61,62]. It includes chromosome separation and encapsulation in a micronucleus, followed by radical rearrangements, potentially leading to an sSMC [63,64]. It should be noted that UPD in connection with inherited sSMCs is extremely rare or does not exist at all [34], whereas de novo sSMCs may carry a high risk of UPD.

As for *de novo* sSMC(15) containing the 15q11–13 region, their clinical severity depends on their DNA content and parental origin. Gene expression in the 15q11–q13 region is regulated by genomic imprinting, which determines exclusive expression from either the maternal or the paternal chromosome [65]. Consequently, ECRs of sSMC(15) of maternal and paternal origin are expected to differ in their manifestations. Indeed, such sSMC(15)s are predominantly of maternal origin [48,49,66]. This is probably the result of either negative selection against paternal gametes containing sSMC or, given imprinting at the 15q11–q13 region, higher mortality of embryos with paternal sSMCs; while carriers of maternally derived sSMCs show a milder and more variable phenotype than expected [52,58,67]. However, due to the small number of reported patients with such paternal sSMCs(15), their clinical relevance remains uncertain. It is also possible that such paternal sSMCs remain underdiagnosed; therefore, very mild or even normal phenotypes of paternal sSMC(15) carriers may exist. 

One of the possible mechanisms that could be involved in idic(15) formation is based on non-allelic homologous recombination (NAHR) and inverted segmental duplication in the proximal region of the long arm of chromosome 15. Results of meiotic recombination depend on the location and orientation of the involved segmental duplications. Formation of idic(15) with BP3:BP3 could be promoted by segmental duplications in opposite orientations in the BP3 region, while homologous segmental duplications in opposite orientations located in the BP4 and BP5 regions could be a prerequisite for asymmetric idic(15) formation. We suppose that such intrinsic personal genomic features could explain the high frequency of idic(15) with BP3:BP3 and BP3:BP5 formations. Elucidating the mechanisms of idic(15) formation mechanisms requires the analysis of BP regions of parental and proband chromosomes at the base-pair level. This would make it possible to reveal specific structural features of the 15q proximal region of the chromosome involved in rearrangement and to identify predisposing parental factors [68].

The proximal region of 22q also shows a very complex and variable organization. The set of eight low-copy repeats (LCRs) makes it one of the most complex regions of human chromosomes [69]. It is characterized by a large variety of deletions, duplications, translocations, and inversions. Rearrangements in the proximal region of 22q are associated with cat eye syndrome (CES), der(22) syndrome, and velocardiofacial/DiGeorge syndrome (VCFS/DGS) [69]. Its various subregions could also be present in sSMC(22). Due to the high complexity of LCRs, the genome assembly of the proximal 22q region remains problematic and requires long-read sequencing and optical genome mapping to unravel its complex duplicon structure.

sSMC(22) comprises two main classes. The first class consists of inverted duplication variants and their derivatives, whereas the second includes translocations involving a small distal region of another chromosome. The sSMCs of the first class typically contain, besides 22p, one or two copies of the proximal 22q region (~2 Mb), known as the cat eye syndrome (CES) critical region [70]. Various bisatellite sSMC(22)s of different sizes, as well as small ring-like SMC(22)s, have also been described [71,72]. The sSMCs belonging to the second class result from a translocation and contain the 22pter–q11 region, along with a small distal region of another chromosome. The der(22)t(8;22)(q24.1;q11.1) leads to der(22)t(8;22)(q24.1;q11.1) syndrome, while der(22)t(11;22)(q23;q11)—to Emanuel syndrome [73] and others. These sSMCs consist of regions derived from two different chromosomes and they will be discussed in the section devoted to complex sSMCs.

Although numerous possible variants exist for sSMC(15) and sSMC(22), for an initial estimation of their DNA content, targeted FISH with locus-specific probes (e.g., *SNRPN* for 15q11–q13, N25/77H2/109L3 for 22q11.2) are widely used [49,74]. In other acrocentric chromosomes (13, 14, and 21), no recurrent breakpoint hotspots comparable to those established for chromosomes 15 and 22 have been revealed. The frequency of sSMCs derived from acrocentrics 13, 14, and 21 is significantly lower, and their DNA content is less predictable. Notably, sSMCs derived from acrocentric chromosomes can undergo additional rearrangements, further increasing their diversity. For instance, data on ring sSMCs derived from acrocentrics have been previously published [33].

Excluding i(5)(p10), i(8)(p10), i(9)(p10), i(12)(p10), i(18)(p10), and i(20)(p10), the formation of sSMCs derived from biarmed chromosomes requires at least two breaks. Linear sSMCs may or may not contain clusters of telomeric repeats on their ends. The origin of these clusters is unclear. Such clusters may be a result of telomerase activity at break sites (*de novo* capping), similar to the formation of the Interstitial Telomeric Sequences (ITSs) observed inside the chromosomes of many vertebrates [75]. However, large deletions with two breaks, one in a proximal region and another in a subtelomeric chromosome region, are considered a possible mechanism [76]. The ring formation of sSMCs represents an alternative to telomere acquisition. Nevertheless, the absence of telomeres may be considered indicative of the sSMC ring morphology; however, it is important to note that linear sSMCs can lack detectable telomeric repeats, while ring sSMCs may contain a detectable cluster of telomeric repeats [33].

Despite the presence of an active centromere, pericentromeric heterochromatic region, and clusters of telomeric repeats, many sSMCs, exhibit mosaicism, suggesting mitotic loss. The mechanisms of sSMC loss remain unclear, as do the processes underlying aneuploidy rescue in some cases. Additional rearrangements during sSMC formation or in later stages of ontogenesis may further increase structural diversity of sSMCs and lead to structural sSMC mosaicism.

## 5. sSMC Derived from Sex Chromosomes

Sex chromosome-derived sSMCs represent a distinct diagnostic entity requiring specific clinical management due to their possible impact on disorders of sex development as well as on the manifestation of X-linked diseases. Here, we consider only supernumerary marker chromosomes derived from the X or Y chromosome in patients with a 47,XX,+mar or 47,XY,+mar karyotype. Derivatives of sex chromosomes in a 46,X,+mar karyotype result in partial monosomies and are not discussed in this review.

X-chromosome-derived sSMCs range from small ring chromosomes in females with Turner syndrome and a mosaic karyotype, such as 46,X,der(X)/47,X,der(X),+r(X) [77] or in males [78] to linear structures with interstitial duplications [79], neocentromeres [80], and complex rearrangements [7]. One of the most important determinants of the clinical effects of these sSMCs is the involvement of the X-inactive specific transcript (XIST) locus (Xq13.2) and the resulting X-chromosome inactivation (XCI) pattern. Generally, the presence of an X-chromosome-derived sSMC with *XIST* in the karyotype is compensated by its epigenetic inactivation. Otherwise, clinical features of X-linked syndromes due to partial trisomy in females or partial disomy in males may appear. Mosaicism for sSMC and cell interference may additionally modify the clinical manifestation, as has been shown for a boy with severe craniofrontonasal syndrome and karyotype mos 47,XY,+r(X)(p11.1q13)/46,XY with a supernumerary ring X chromosome containing the *EFNB1* gene responsible for this disorder, but lacking *XIST* [81].

Y chromosome-derived sSMCs frequently occur as small rings, inverted duplications, or minute linear fragments. sSMCs derived from the Y chromosome with a neocentromere formation have also been reported both in a newborn male with a normal phenotype [82] and in a patient with azoospermia [83]. Evaluation requires testing for the sex-determining region Y (*SRY*) gene and pericentromeric Y heterochromatin. Phenotypic manifestations range from normal male fertility to ambiguous genitalia and mixed gonadal dysgenesis. Crucially, the presence of Y-derived chromosome material in females with gonadal dysgenesis confers a ~15–30% risk of developing gonadoblastoma, necessitating counseling regarding prophylactic gonadectomy [84].

## 6. Complex, Multiple, Ring, and Neocentric sSMCs

Complex sSMCs consist of chromosomal material derived from more than one chromosome, featuring at least one breakpoint located in the proximal region of the q-arm of an acrocentric chromosome and another in a distal region. As mentioned above, some of these markers result from various translocations with other chromosomes. Approximately 9% of sSMCs derived from chromosome 22 are der(22)t(8;22)(q24.1;q11.1), which is associated with der(22)t(8;22)(q24.1;q11.1) syndrome. The supernumerary der(22)t(11;22)(q23;q11) chromosome originated from a common recurrent reciprocal translocation, and this sSMC is associated with Emanuel syndrome [73]. The breakpoint regions at 22q11 and 11q23 are enriched for palindromic AT-rich repeats that form hairpin/cruciform structures, which mediate double-strand breaks during meiosis, thereby increasing the frequency of rearrangements in these regions [25]. More than half of complex sSMCs result from parental balanced translocations. They contain gene-rich subtelometic regions, and their presence typically leads to severe clinical consequences. The mechanism underlying the formation of similar complex sSMCs involving the centromeric regions of biarmed chromosomes remains poorly understood.

Complex sSMCs consist of the proximal region of one chromosome and small region derived from the distal region of an arm of another chromosome [1]. They are usually enriched in genes and should be reliably distinguished from the inverted duplications described above. For example, at the first diagnostic step, both inv dup(22) leading to CES and der(22)t(11;22)(q23;q11) leading to Emanuel syndrome showed the centromeric region of chromosome 22. At the next step, they should be reliably differentiated. For example, two FISH signals on the marker chromosome obtained with a DNA probe specific to the 22q11 region and no signal from the labeled DNA of chromosome 11 suggest inv dup(22), whereas two FISH signals on the sSMC, one specific to 22q11 and the other to 11q23, indicate der(22)t(11;22). Application of aCGH would reveal a copy number gain involving exclusively the proximal long arm of chromosome 22 (22q11.1–q11.21) in the case of inv dup(22), while two distinct genomic imbalances in 22q11 and 11q23-qtel would be detected in the case of der(22)t(11;22). Notably, the 11q23–qtel region in the der(22)t(11;22) could remain undetected by 24-color FISH due to its small size. 

sSMCs of complex composition could also arise from multiple chromosomal rearrangements. Usually, they contain discontinuous segments from the original or additionally several other chromosomes [7,11,25]. High-resolution analysis of ten such sSMCs performed at the base-pair level and combined with the T2T-CHM13 assembly enabled precise mapping of BPJs and a detailed description of their discontinuous ECRs consisting of regions derived from the original and other chromosomes [7]. Based on the obtained data, molecular mechanisms of sSMC formation, including microhomology-mediated end joining (MMEJ) and microhomology-mediated break-induced replication (MMBIR), have been suggested. The involvement of numerous chromosomes in complex sSMC formation points to chromothripsis [85] and chromoanasynthesis [86], followed by breakage–fusion–bridge cycles. Furthermore, haplotype analysis indicated trisomy rescue in several investigated cases [7].

Another type of sSMC is multiple sSMCs differing in origin and composition, which present an additional diagnostic challenge. We propose that even identical ECR content of sSMCs may lead to different phenotypic outcomes, depending on whether their ECRs reside on a single “large” sSMC or are distributed across multiple small sSMCs. The microenvironment for ECRs in one large or multiple sSMCs differs: sSMCs with small ECRs consisting mainly of heterochromatin are likely located in the nuclear periphery in the transcriptionally inactive B compartment of the interphase nucleus. This position in the nucleus could lead to their gene silencing or at least partial suppression of gene transcription. The hypothesis regarding the potential significance of the ECR size of a marker chromosome and its localization within interphase nuclear compartments requires experimental verification. This is especially true given that some observations do not align with it [28]. In the diagnosis of any sSMC, all data regarding its origin, the gene content of its ECR (euchromatic region), sSMC copy number, the regulation of the genes it contains, and mosaicism in the relevant tissues must be taken into account. The significance of the ECR size and sSMC location in the interphase nucleus remains an open question [87]. The influence of sSMC ECR size and its location in different nuclear compartments on sSMC manifestation remains purely speculative, and these sSMC features cannot serve as a diagnostic criterion for assessing sSMC pathogenicity.

The ring morphology of the sSMC might influence its location in the nucleus and could increase rsSMC instability. Mitotic breakage and subsequent imperfect repair in cell cycles can lead to different sSMC copies according to their size and DNA content. Indeed, sequencing of the microdissected DNA library obtained from individual rsSMC copy revealed small deletions in its ECR [28]. aCGH or WGS of DNA isolated from thousands of cells attribute all detected additional DNA fragments to sSMC, without taking into account the actual ECR size and content of individual rsSMC copies. Studies of rsSMCs with lrGS combined with T2T-CHM13 assembly have also revealed the complex, discontinuous nature of their ECRs [7]. Regarding phenotypic effects, it is plausible that a more compact rsSMC conformation favors B-compartment localization and that transcriptional suppression of rsSMC genes may consequently modify rsSMC manifestation. For example, an rsSMC with a ~10 Mb ECR and numerous OMIM-associated genes was unexpectedly found to have no pathogenic effect [28]. To better understand the phenotypic features of rsSMCs, further studies are needed to elucidate the mechanisms underlying their phenotypic effects and to propose new criteria for evaluating sSMC pathogenicity. At present, the significance of the sSMC location in the A (active) or B (inactive) compartment of the interphase nucleus remains a hypothesis.

sSMCs with neocentromeres represent a rare sSMC variant. We speculate that, due to the lack of a pericentromeric heterochromatic region, these neocentromeric sSMCs (neo-sSMCs) could be located in the A compartment of the interphase nucleus, and their clinical manifestation could resemble that of a typical segmental duplication of the identical region on the intact homolog. To date, no data have been published to support this assumption, which remains purely hypothetical.

**Figure 1:**
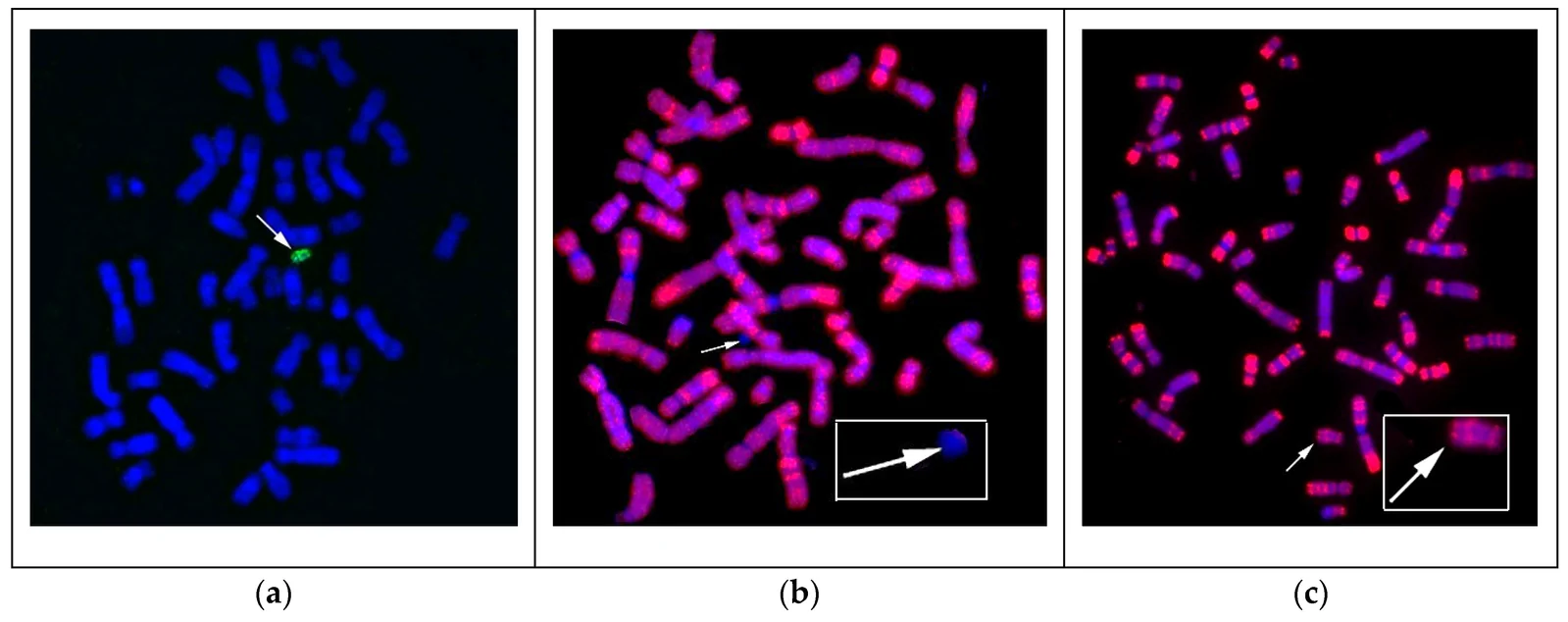
Fluorescence *in situ* hybridization (FISH) on metaphase chromosomes of a patient with a marker chromosome. (**a**) FISH using a microdissected DNA probe derived from a small supernumerary marker chromosome (sSMC), which contains a neocentromere and a region of unknown origin (green). The patient’s karyotype is 48,XY,+21,+mar. (**b**) FISH using an Alu-repeat probe labeled in red on marker chromosomes without an euchromatic region, and (**c**) the same probe on metaphase chromosomes, including the sSMC with an ER. DNA was counterstained with DAPI (blue). Arrows indicate the sSMCs.

Notably, sSMCs with neocentromere and an ECR of unidentifiable origin have been described previously in prenatal diagnostics [40] (Figure 1a); microdissection and FISH confirmed their sSMC status, but pathogenicity remained undetermined due to concurrent trisomy 21 and pregnancy termination.

## 7. Application of Molecular Cytogenetic and Cytogenomic Techniques in the sSMC Diagnostics

Typically, the first step in the diagnosis of sSMCs following their detection on metaphase spreads is to determine their origin. However, most familial sSMCs that lack an ECR also contain no genes and do not result in a genetic imbalance or UPD [34]. One would expect them to have no pathogenic effect, or at least a very mild one. In the case of such an sSMC, attention should be paid to confirming the absence of genes within the marker chromosome, as well as to excluding any structural mosaicism that might mask a genetic imbalance present in some tissues.

In our view, detecting the presence of an ECR in an sSMC is of greater importance. In this regard, analysis of an sSMC for the presence of an ECR in cases involving an inherited sSMC may be most useful as an initial diagnostic step following the detection of an sSMC in metaphase spreads. ECRs of an sSMC that include R-band(s) may be revealed with replicative staining [88], but small R-subbands may escape detection with this staining technique. FISH using a labeled Alu-repeat probe has proven to be more sensitive, painting ECRs while leaving heterochromatin regions unstained. In addition to ECRs, this approach marks only a small, dot-like region enriched for Alu repeats within the satellites of human acrocentric chromosomes [89]. This specific distribution pattern in the human genome enables the application of Alu-repeat FISH for ECR visualization within sSMC [90], allowing for a more reliable differentiation between heterochromatic (Alu-negative; Figure 1b) and euchromatic (Alu-positive; Figure 1c) chromosomal regions compared to replicative banding. Alu-positive sSMCs contained ECRs [91]. Although preparing the Alu-repeat probe requires no special equipment [89,90], its application does not always allow for a clear classification of all analyzed sSMCs as either ECR-positive or ECR-negative. Some sSMCs exhibiting a weak, near-background Alu FISH signal require further investigation using alternative techniques. Determination of the sSMC origin can be achieved via FISH with pericentromeric-specific probes or sSMC-derived microdissected DNA probes [19,21,92]; FISH with the corresponding locus-specific DNA probes can be deployed to detect small ECRs within the sSMC.

**Figure 2:**
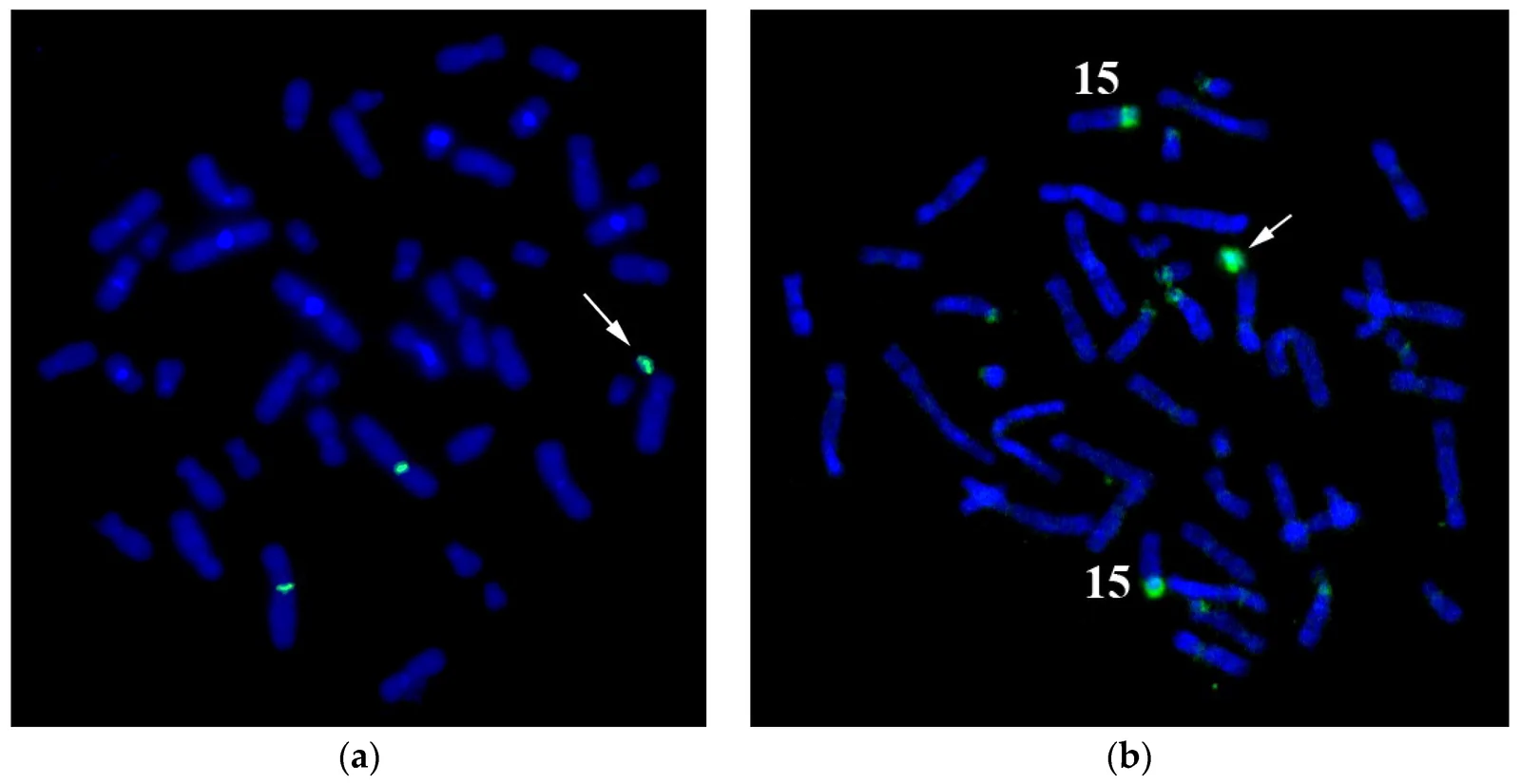
FISH with microdissected DNA probes (green signal). (**a**) The painting probes were generated from the sSMCs, which consist of the proximal region of chromosome 2 (sSMC(2)) and (**b**) chromosome 15 (sSMC(15)). The DNA was counterstained with DAPI (blue). Arrows indicate the sSMCs.

Multiplex (24-color) FISH specifically paints euchromatic regions of different chromosomes and can determine ECR origin. This technique is recommended to confirm the identity of inverted duplications affecting the short arms of submetacentric chromosomes or chromosome 20, which are initially detected via GTG- or DAPI-banding (Figure 2a). Unfortunately, 24-color FISH identifying the chromosome origin of ECR in the sSMC cannot identify the region inside the chromosome [20,41,42]. Furthermore, 24-color FISH shows lower sensitivity for small ECRs in comparison with FISH using locus-specific DNA probes and even painting performed with WCP for original chromosome. It should be noted that chromosome painting including 24-color FISH does not paint C-positive chromosome regions, thus providing no information on sSMCs containing no ECR [20,41,42]. CISS-hybridization with microdissected DNA probes overcomes this problem by painting both euchromatic and heterochromatic regions of original chromosome (Figure 2a), due to the increased quantity of repetitive DNA specific to the original C-positive region. Suppression of repetitive sequences hybridization used in the standard CISS-hybridization protocol appears to be incomplete in the original C-positive region leading to its specific painting. Microdissected DNA probes obtained from sSMCs derived from two-arm chromosomes do not paint the C-positive regions of other chromosomes, while microdissected DNA probes obtained from sSMCs derived from acrocentrics besides original C-positive region slightly paint C-positive regions of other acrocentrics complicating original chromosome identification (Figure 2b).

The resolution of FISH with microdissected DNA probes on metaphase or prometaphase chromosomes is limited to the identification of chromosome bands or subbands. More precise mapping of ECR borderlines requires aCGH, WGS or FISH with locus-specific DNA probes [93,94,95,96]. However, aCGH is unreliable in cases of low-level sSMC mosaicism [96,97] and cannot distinguish between multiple sSMCs and a single complex sSMC containing ECRs present in multiple sSMCs [11]. FISH with a microdissected sSMC probe obtained from single sSMC copies resolves this problem [11,28]. lrGS combined with read mapping to T2T-CHM13 assembly could potentially be exceptionally powerful, achieving base-pair BPJ resolution even in low-frequency mosaics; however, accuracy was lower in regions enriched for repetitive DNA [7,9,12]. We suppose that multiple sSMCs and heterogeneous sSMC copies may complicate sSMC assembly performed with this approach. The first studies of sSMCs using lrGS and OGM offer promise for the successful application of these technologies, even in cases of sSMCs that are challenging for diagnostics. Further research will allow a more precise assessment of the capabilities and limitations of their use, and will help determine their performance quality according to mosaicism level, repetitive sequence content, structural heterogeneity, and the required sequencing depth.

**Figure 3:**
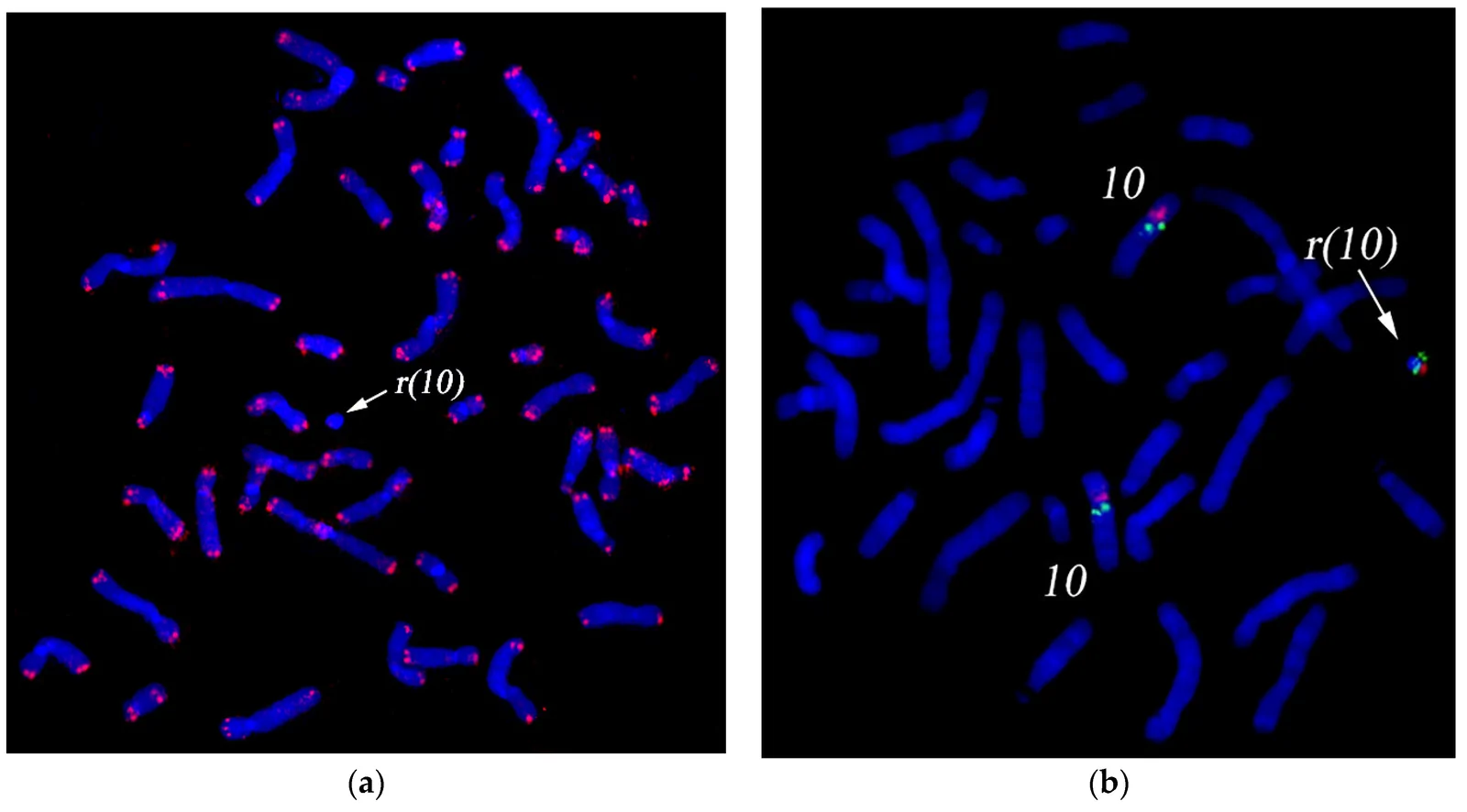
FISH analysis of metaphase chromosomes from a patient with a ring sSMC(10). (**a**) FISH using a telomere-specific DNA probe (red signals). (**b**) FISH using locus-specific probes *ZNF248* (10p11.21, red) and *MAPK8* (10q11.22, green). On the normal chromosome 10, the signals were localized separately on the p and q arms. On the ring sSMC(10), however, the signals were colocalized, confirming the ring structure of the rSMC. Arrows indicate the ring chromosome r(10)(p11.21;q11.23). DNA was counterstained with DAPI (blue).

Ring morphology can be suggested in cases of telomeric FISH signal absence (Figure 3a), but some rsSMCs have shown FISH signals from labeled telomeric repeats. FISH with locus-specific probes flanking the expected breakpoints could confirm ring morphology by showing colocalization of FISH signals on rsSMCs but separate signals on normal chromosomes (Figure 3b). Complex rsSMCs require lrGS application for their accurate reconstruction [7,9,12].

Additional specific genetic testing should be considered for sSMCs derived from sex chromosomes, including the assessment of X-chromosome inactivation status and the presence of the *SRY* gene.

## 8. Proposed System for Human sSMC Classification

The optimal approach to sSMC characterization depends on its specific type. This prompts us to propose a new framework for the description of sSMCs, centered on the clinical and diagnostic questions that arise following sSMC detection. The most recent classification divides sSMCs into five main classes [23], categorized according to a combination of three key features: (a) derivation from acrocentric or non-acrocentric chromosomes; (b) the presence of chromosome-specific α-satellites; and (c) the structural rearrangement of the SMC. Class 1 includes the majority of SMCs that originate from acrocentric chromosomes, containing chromosome-specific α-satellites, and feature continuous or discontinuous ECRs from the chromosome of origin. Class 2 consists of SMCs originating from non-acrocentric chromosomes that contain chromosome-specific α-satellites without detectable additional rearrangements. Class 3 includes SMCs originating from non-acrocentric chromosomes that contain chromosome-specific α-satellites and exhibit rearrangements involving regions of the chromosome of origin, including isodicentrics or rings. Class 4 consists of sSMCs originating from non-acrocentric chromosomes that contain chromosome-specific α-satellites and display additional regions originating from a chromosome other than the sSMC itself. Class 5 includes neocentromeric sSMCs that lack chromosome-specific α-satellites [23]. While an obvious advantage of this classification is that any given sSMC can be unambiguously assigned to a single class, sSMCs that require fundamentally different diagnostic workflows are often grouped together, which complicates its application in routine clinical diagnostics.

We propose a classification based on the specific features of sSMCs—such as their pathogenicity, size, DNA content, morphology, and mechanism of origin. This enables different sSMC types to be distinguished, thereby guiding the selection of appropriate diagnostic approaches and techniques. Undoubtedly, entirely heterochromatic sSMCs (lacking ECRs) and sSMCs containing ECRs require fundamentally different analytical methods to evaluate their pathogenic potential. Entirely heterochromatic sSMCs derived from different chromosomes may consist of a centromere and pericentromeric heterochromatin, and they may or may not contain telomeric repeat clusters; however, none of them contain functional genes or lead to genetic imbalance. Consequently, these sSMCs are expected to be benign. However, when they arise *de novo*, their occurrence might be associated with the UPD of the chromosome of origin, causing imprinting disorders in the case of sSMCs derived from chromosomes 6, 7, 8, 11, 14, 15, or 20. As a result, following the detection of *de novo* entirely heterochromatic sSMCs, the most critical objectives are confirming the absence of ECRs and identifying the chromosome of origin to perform subsequent UPD testing in case of sSMC derived from chromosomes 6, 7, 8, 11, 14, 15, or 20.

sSMCs with ECRs exhibit more diverse clinical manifestations as a result of the underlying genetic imbalance, requiring diagnostic methods to precisely determine their ECR content. However, sSMCs consisting of the short arms of chromosomes 5, 8, 9, 12, 18, and 20 lead to tetrasomy or trisomy involving large ECRs comparable in size to an entire small autosome. All of them are pathogenic or highly pathogenic, although phenotypic manifestations might be milder in cases with low mosaicism levels in the affected tissues [1]. Even the smallest of them, i(18p), leads to tetrasomy 18p syndrome [98]. Consequently, this specific group of sSMCs warrants separate consideration. The additional rearrangements occurring within the extrachromosomal euchromatic material of these sSMCs may provide insights into the mechanisms of chromosomal restructuring, but if they involve only a small region, these rearrangements should probably not alter the overall conclusion regarding sSMC pathogenicity.

sSMCs derived from chromosomes 15 and 22 constitute the majority of all sSMCs and differ from others in their mechanism of origin. They likely arise via crossing-over within inverted regions and subsequent breakage-and-fusion events at specific breakpoint hotspots in the long chromosome arms. Consequently, the majority of sSMC(15) and sSMC(22) markers represent a limited set of variants regarding the DNA composition of their ECRs. This predictability has enabled the development of DNA probes specifically designed to test for the presence of critical pathogenetic regions within these sSMCs. However, they may also undergo additional rearrangements that alter their morphology and ECR content. Furthermore, sSMCs of maternal and paternal origin derived from these chromosomes can differ phenotypically due to imprinting within the proximal region of 15q. Consequently, their pathogenicity often depends on parental origin, necessitating the clarification of this factor. For many variants of these sSMCs, the potential pathogenicity has already been evaluated, making detailed analysis of ECR content increasingly important. Besides the recurrent breakpoint hotspots located in the proximal regions of chromosomes 15 and 22, there are recurrent breakpoint hotspots in distal chromosome regions. The involvement of both types of hotspots could lead to complex sSMCs consisting of regions derived from two different chromosomes, such as the aforementioned der(22)t(8;22)(q24.1;q11.1) and der(22)t(11;22)(q23;q11). Thus, identifying the chromosome of origin as 15 or 22 does not rule out a complex sSMC nature.

To date, recurrent breakpoint hotspots in the proximal ECRs of other chromosomes have not been identified, rendering the ECR content of sSMCs derived from them less predictable. Usually, they consist of variable proximal regions of the involved chromosomes, with or without telomeric caps. It should be noted that the absence of detectable clusters of telomeric repeats in an sSMC suggests, but does not guarantee, a ring morphology; vice versa, a FISH signal from labeled telomeric repeats does not exclude a ring morphology [33]. Additional sSMC rearrangements after their onset (deletions, duplications, or transpositions) can result in sSMCs with discontinuous ECRs. Alternatively, chromothripsis [85] and chromoanasynthesis occurring during sSMC formation could lead to an initial sSMC with discontinuous regions derived from different chromosomes. Despite the complex organization of such sSMCs, modern cytogenomic techniques, including aCGH and lrGS, allow for their detailed description. While aCGH determines the copy number of ECRs in the patient’s genome without identifying their chromosomal localization, the application of lrGS enables the generation of T2T sSMC assemblies [7,9]. Unfortunately, evaluating the pathogenicity of an sSMC with numerous discontinuous regions remains an even more challenging task.

Of particular interest are sSMCs that carry a neocentromere instead of the centromere of the chromosome of origin. Their origin cannot be determined via FISH using DNA probes specific to pericentromeric regions, and their characterization requires specialized methods, such as generating a microdissected DNA library followed by high-throughput sequencing or using microdissected probes for FISH. It is reasonable to expect that the pathogenicity of a neocentromeric sSMC (neo-sSMC) would be similar to that of a segmental duplication of the homologous region on the intact original chromosome. However, possible epigenetic changes and increased mitotic instability of the neo-sSMC must be taken into account.

Based on sSMC features that are critical for determining diagnostic strategies and evaluating pathogenicity, sSMCs can be classified into several main groups and subgroups:

1. Heterochromatic sSMCs consisting of a centromere and pericentromeric heterochromatin, lacking ECRs, and containing or lacking telomeric repeat clusters.
  - Familial sSMCs—likely benign.
  - *De novo* sSMCs—require UPD testing of the original chromosome if that chromosome is 6, 7, 8, 11, 14, 15, or 20. The UPD of these chromosomes has clinical manifestations.
2. sSMCs containing ECRs (*de novo* occurrence of sSMC derived from chromosomes is 6, 7, 8, 11, 14, 15, or 20 requires UPD testing of the original chromosome). Besides the possible imprinting effect, uniparental isodisomy (two identical copies of one chromosome homolog from one parent) leads to homozygosity for all recessive pathogenic gene variants located on the chromosome:
  - Pathogenic or highly pathogenic sSMCs consisting of the short arms of chromosomes 5, 8, 9, 12, 18, and 20; pathogenicity depends on somatic mosaicism pattern.
  - sSMCs derived from chromosomes 15 and 22; predominantly inverted duplications presenting with several ECR variants. Pathogenicity depends on the specific ECR defined by the involved breakpoint hotspot, potential additional rearrangements, and the mosaicism pattern.
  - Linear sSMCs consisting of the proximal ECR of the chromosome of origin, with or without telomeric caps; derived from biarmed chromosomes or acrocentrics 13, 14, and 21. Characterized by variable ECR sizes; pathogenicity depends on ECR size, gene content, and the mosaicism pattern.
  - sSMCs containing discontinuous segments derived from the chromosome of origin or multiple chromosomes, requiring a comprehensive multi-platform cytogenomic analysis.
  - Multiple sSMCs of diverse chromosomal origins and compositions.
  - Ring sSMCs prone to mitotic instability and size variation, which may or may not contain telomeric repeat clusters; they sometimes exhibit mild pathogenicity or can even be benign despite their large size and rich gene content.
  - Neocentric sSMCs lacking pericentromeric heterochromatin with unclear pathogenicity (is their manifestation similar to homologous segmental duplications in the original chromosome?).
  - sSMCs derived from sex chromosomes, which often structurally represent deleted or rearranged fragments of the chromosome of origin.

Unlike the previous sSMC classification [23], this set of sSMC groups and subgroups takes into account more features that are critical for diagnostics and the interpretation of results. On the other hand, a given sSMC could be assigned to more than one subgroup. While an ideal sSMC classification should probably be multidimensional, such an approach would be too complicated for routine practical use. We assume that currently, rather than focusing strictly on classification, the primary emphasis should be placed on the precise and comprehensive description of each sSMC. This description must incorporate all information on sSMC characteristics essential for determining potential pathogenicity, namely: chromosomal origin (the origin of the pericentromeric region or the identification of a neocentromere, and the determination of parental origin); familial or *de novo* status; sSMC mosaicism patterns across all studied tissues; ECR status (positive or negative); and a detailed description of the regions present within the sSMC (including region identification, ECR content, the continuous or discontinuous nature of the detected ECRs, their copy number, and their precise structural order, up to a T2T assembly). Furthermore, it should include the overall sSMC structural organization, potential structural diversity among sSMC copies, and whether it occurs as a unique sSMC or as part of multiple sSMCs. Additional specific genetic testing should be considered for sex chromosome-derived sSMCs, including the assessment of X-chromosome inactivation status and the presence of the *SRY* gene.

The optimal diagnostic strategy for sSMCs and the selection of appropriate methods depend heavily on the specific sSMC types. In our view, incorporating this aspect to characterize sSMCs based on the aforementioned features can facilitate a truly comprehensive diagnosis.

**Figure 4:**
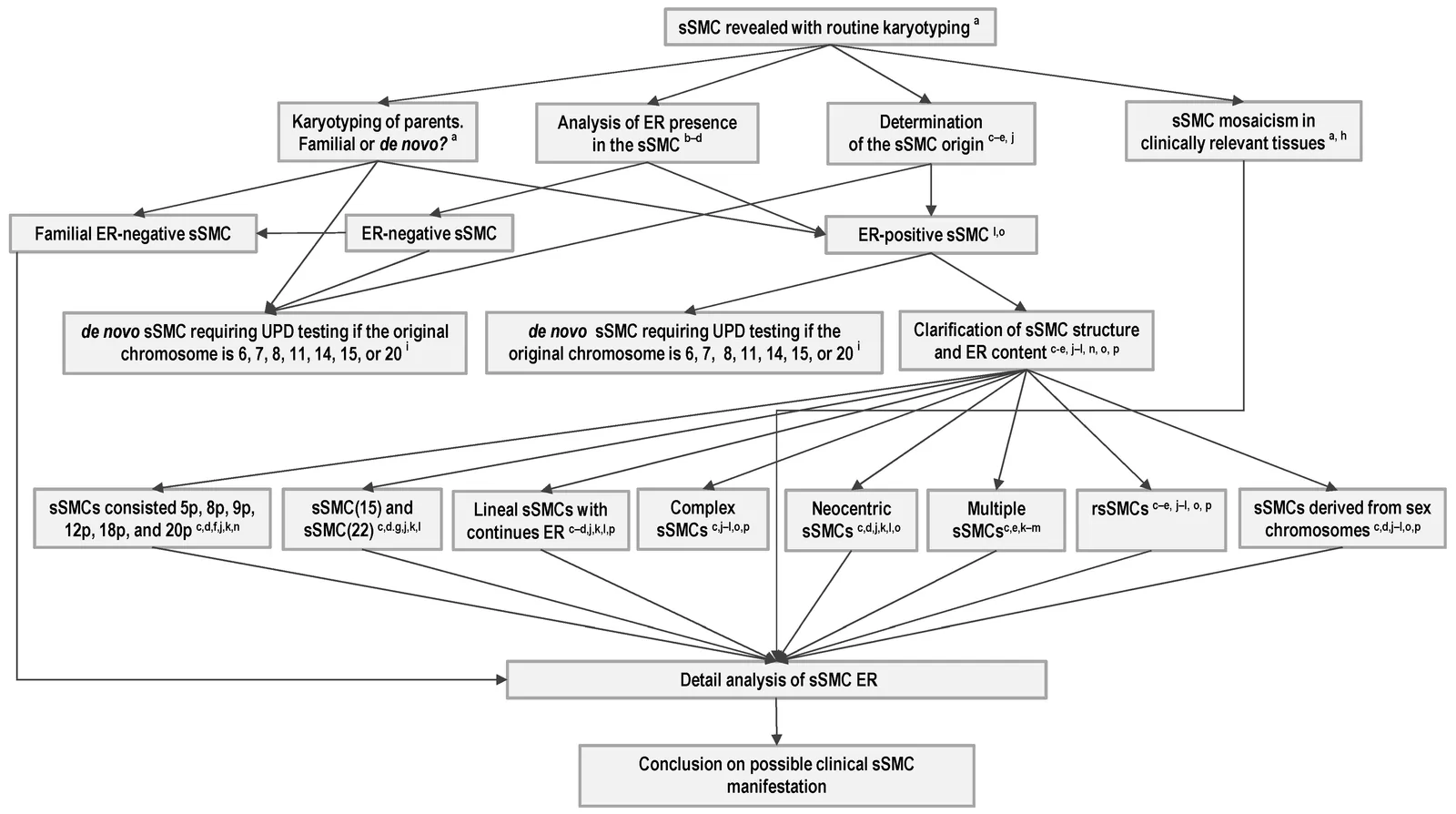
Diagnostic workflow for the characterization and classification of sSMCs: (a) routine karyotyping; (b) testing for the presence of ECRs; (c) chromosome painting using WCPs/ACPs; (d) FISH using sSMC-derived DNA probes; (e) FISH with DNA probes specific to pericentromeric regions; (f) GTG/DAPI-banding; (g) Ag-NOR banding; (h) interphase FISH with locus-specific DNA probes; (i) UPD testing in cases where the UPD of the original chromosome has clinical manifestations (chromosomes 6, 7, 11, 8, 14, 15, or 20); (j) aCGH and WGS; (k) NGS of microdissected sSMC DNA libraries; (l) FISH with DNA probes specific to the region of interest; (m) FISH using microdissected DNA probes generated from individual sSMC copies; (n) multicolor chromosome banding (mBAND); (o) FISH with telomeric probes; (p) T2T sSMC assembly.

Data on mechanisms of sSMC formation, recommended diagnostic techniques for sSMCs of various types within the proposed classification, the informational value of techniques used for sSMC analysis, and possible approaches for pathogenicity estimation are presented in Table 2 and Figure 4. The final conclusion regarding the possible clinical manifestations of sSMCs should be based on the integration of all available data on genomic content, dosage sensitivity, copy number, mosaicism, mode of inheritance, relevant UPD/imprinting findings, breakpoint effects, and additional genomic imbalances.

The development of new technologies such as optical genome mapping, Hi-C, 3D-FISH, and epigenomic profiling holds promise for elucidating sSMC spatial organization, sSMC impact on the spatial organization of the interphase nucleus, the structure of topologically associated domains in all chromosomes, and gene transcriptional activity. While not yet routine, integrating iPSC-derived models and single-cell multi-omics may revolutionize sSMC pathogenicity assessment by capturing tissue-specific mosaicism and dynamic gene regulation [99]. Today, these technologies are deployed to address fundamental questions concerning the nature of sSMCs, including the mechanisms of their biogenesis, structural alterations during ontogenesis, the establishment of mosaicism, gene expression profiles, and their overall impact on the carrier phenotype. The insights gained from these studies are expected to drive the development of novel approaches in sSMC diagnostics.

## 9. Conclusions

Accurate diagnosis of sSMC requires a reliable, accessible, and multi-tiered approach. lrGS combined with telomere-to-telomere (T2T) reference genome assemblies represents a powerful emerging tool for sSMC characterization, complementary to other diagnostic approaches for the description of complex sSMCs. Unfortunately, its cost and the need for appropriate laboratory infrastructure could limit its widespread adoption. A strategic combination of classical cytogenetics, FISH, chromosome microdissection, aCGH, and targeted sequencing remain essential. The selection of diagnostic algorithms must be sSMC-type-dependent, prioritizing ECR detection, gene content, precise ECR borderline mapping, sSMC morphology clarification, and sSMC mosaicism assessment across available tissues. In the future, one possible approach for assessing sSMC pathogenicity could involve considering the sSMC ECR composition—alongside an analysis of similar constitutional microduplications—by adapting the ACMG/ClinGen guidelines for the analysis of copy number variations. However, the adaptation of the ACMG/ClinGen guidelines proposed here is a conceptual framework for future development rather than a validated diagnostic algorithm. It is nonetheless clear that this will be complicated by the unique characteristics of sSMCs, such as their mosaicism, mitotic instability, and three-dimensional nuclear localization. Notably, comparison of inv dup(15) containing the 15q11–q13 region with interstitial duplication of this region revealed differences in their manifestations. It should be noted that inv dup(15)(ptel-q13) differed from the interstitial duplication of the 15q11–q13 region in the location and copy number of this region [59]. Although the integrated use of current cytogenetic and cytogenomic technologies significantly overcomes the limitations of individual methods, challenges in analyzing numerical and structural sSMC mosaicism, combined with difficulties in data interpretation, often prevent a definitive conclusion regarding sSMC pathogenicity.

We suggest that, to advance sSMC diagnostics, future research should prioritize single-cell multi-omics, spatial transcriptomics, and functional assays in iPSC-derived lineages to bridge the gap between genomic architecture and clinical phenotypes. Until standardized, high-precision workflows become universally available, collaborative networks and shared databases will remain vital for refining genotype–phenotype correlations and improving genetic counseling.

## Figures and Tables

**Table 1:** Sixty-five-year timeline of sSMC studies (1961–2026).

Year	Milestone/Technological Breakthrough	References
1961	First sSMC identified by routine metaphase chromosome staining	[6]
1985	Identification of sSMC(18p) via radioactive *in situ* hybridization	[14]
1986	Cat eye syndrome linked to sSMC(22)	[15]
1987	First report of a patient with extreme variation in number and size of sSMCs	[16]
1990	First centromeric repeat FISH; CISS with sorted chromosome libraries	[17,18]
1992	Flow sorting with reverse painting	[13]
1994	sSMC microdissection with DOP-PCR	[19]
1997	Spectral karyotyping (SKY) applied to sSMC origin	[20]
2001–2004	Multicolor FISH panels (cenM-FISH, subcenM-FISH, MCB)	[21,22]
2003	First systematic review and classification of sSMCs	[23]
2005	Public online ChromosOmics Database launched	[1]
2007	High-density pericentromeric BAC array CGH for mosaic sSMCs	[24]
2008–2014	Detailed analysis of complex sSMCs; SNP arrays applied for sSMC delineation and UPD detection in prenatal diagnosis	[3,25,26]
2019	Preimplantation genetic testing for structural rearrangements (PGT-SR) with NGS for sSMC carriers	[27]
2022	Targeted long-read sequencing with adaptive sampling; first neocentric, complex sSMC characterized by OGM	[9,11]
2026	T2T assembly for complex sSMC	[7]

**Table 2:** Classification, Structure, and Pathogenicity of sSMCs.

Type of sSMC	Specific DNA Content and Structural Organization	Origin Mechanisms	Inheritance	Criteria for Estimation of Pathogenicity	Comments
sSMCs without euchromatin (pericentromeric heterochromatin)	Pericentromeric heterochromatin (C-positive); novel telomeric repeat clusters at ends; absence of euchromatin	Breakage–fusion events; telomerase-mediated healing; meiotic/mitotic segregation errors	*de novo* and inherited variants	Familial sSMCs are likely benign variants without clinical manifestations. Reproductive outcomes for carriers may be affected.	Familial sSMC impact on phenotype is similar to normal heterochromatin variants (chr 1, 9, 16, Y)
				*De novo* sSMCs—risk of UPD of the original chromosome if that chromosome is one of 6, 7, 8, 11, 14, 15, or 20. The UPD of these chromosomes has clinical manifestations.	UPD testing is recommended for *de novo* cases if that chromosome is one of 6, 7, 8, 11, 14, 15, or 20. The UPD of these chromosomes has clinical manifestations.
sSMCs derived from the short arms of submetacentric chromosomes (i(5p), i(8p), i(9p), i(12p), i(18p)), and i(20p).	One or two copies (usually inverted duplication) of short arms; partial tri- or tetrasomy of euchromatin	U-type exchange; Inverted duplications of the short arms	Predominantly *de novo*	Pathogenic variants due to partial trisomy or tetrasomy. Specific syndromes: tetrasomy 5p syndrome (often found in a mosaic state); mosaic tetrasomy 8p; tetrasomy 9p syndrome; Pallister–Killian mosaic syndrome; isochromosome 18p syndrome; tetrasomy 20p/trisomy 20p syndrome. Phenotypic manifestations depend on mosaicism level in the affected tissues.	Expressivity strongly depends on the mosaicism level. Mosaicism assessment in multiple tissues is critical (e.g., 12p sSMC: low in blood, high in fibroblasts). *In vitro* cultivation may distort frequency via clonal selection
sSMCs derived from acrocentric chromosomes 15 and 22 (breakpoint hotspots)	Inverted duplication of short arm and proximal long arm; two centromeric regions (one inactive); often two NORs	NAHR between LCRs at breakpoint hotspots; U-type exchange; predisposition by parental pericentric inversions	Usually *de novo*, but may be predisposition due to pericentric inversions	Usually pathogenic variants, but depends on the size of duplication/triplication and the number of involved genes. Proximal tetrasomy 15q, cat eye syndrome (22q11.2) correlate with increased gene dosage. Derivatives of chromosomes 15 and 22 should be reliably differentiated from complex sSMCs containing a region derived from another chromosome.	Most common sSMC type. Breakpoint mapping is essential for prognosis. Two NORs are characteristic but size varies; secondary constrictions may be invisible.
sSMCs consisting of proximal euchromatic regions with telomeric caps (metacentric/submetacentric, chr 13, 14, 21)	Centromere and proximal region (hetero-/euchromatin) and telomeric repeat clusters at ends. For 13, 14, 21: inv dup of p-arm and proximal q-arm	Chromosome breakage and repair; neotelomere formation; U-type exchange; possible large deletions followed by telomerase healing	Predominantly *de novo*	The size of the ECR cannot serve as a definitive criterion for the estimation sSMC pathogenic potential.	DNA composition is less predictable for chr 13, 14, and 21 (no established hotspots). UPD testing is recommended for *de novo* sSMC(14). Telomeric clusters indicate structural stability.
Complex derivatives (discontinuous segments from original or several chromosomes)	Discontinuous/non-colinear regions of original chromosome; chimeric segments from multiple chromosomes; microhomology-mediated junctions	Balanced translocation of a parental chromosome. Chromothripsis, chromoanasynthesis, breakage–fusion–bridge cycles (BFB), microhomology-mediated end joining (MMEJ), microhomology-mediated break-induced replication (MMBIR), complex translocations; possible trisomy rescue events	Predominantly *de novo*	An sSMC derived from a balanced translocation of a parental chromosome is associated with the described syndromes. Other sSMCs could show highly variable manifestation depending on the transcriptional activity of the genes they contain.	Requires reliable differentiation from sSMCs of different types. Requires integrated multi-omics/cytogenomics workflow. lrGS is currently the high standard for breakpoint resolution. Mosaicism and structural complexity complicate prognosis
Multiple sSMCs of different origins and composition	Two or more distinct sSMCs per cell or across different cell clones; varying euchromatic/heterochromatic content	Independent formation events; genomic instability; sequential marker accumulation; possible chromothripsis or replication stress	Predominantly *de novo*	Cumulative/additive effect; evaluated individually per marker. Depends on the gene dosage of each sSMC	Mosaic distribution across clones is common. Requires clone-specific analysis. Phenotypic prediction is challenging due to additive/epistatic effects and variable tissue distribution.
Ring sSMCs	Centromere with proximal euchromatin/heterochromatin; end-to-end fusion; lacks telomeric repeats; may contain discontinuous or deleted segments	Terminal deletions and end-to-end fusion; BFB cycles; mitotic breakage-repair; somatic instability	Predominantly *de novo*	Ring morphology could lead to somatic deletions increasing instability and sSMC diversity.	Less mitotically stable than linear sSMCs. Size by aCGH may be overestimated. Requires FISH validation for ring structure and breakpoint mapping. UPD testing is recommended in the case of *de novo* ring sSMCs derived from chromosomes 6, 7, 8, 11, 14, 15, or 20. The UPD of these chromosomes has clinical manifestations.
Neocentric sSMCs	Functional neocentromere (epigenetically activated, lacks alpha-satellite DNA), euchromatin, telomeric repeats; may contain unknown DNA/Alu repeats	Epigenetic centromere formation on non-centromeric chromosome region; chromosomal breakage and repair; telomerase healing; possible replication stress	Predominantly *de novo*	The expected pathogenicity of these markers could be similar to that of a homologous segmental duplication within an intact chromosome.	Rare variant. Cases of unknown origin may contain Alu repeats. Exclusion of segmental duplications of original chromosome is required. Epigenetic nature complicates prediction.
sSMCs derived from sex chromosomes	Different types, including ring sSMCs are possible	Meiotic/mitotic segregation errors, concomitant UPD formation is reported	Usually *de novo*	For X-chromosome-derived sSMCs, the expression of *XIST* and subsequent X-chromosome inactivation are major determinants of whether the marker exerts a benign or pathogenic effect. For Y-chromosome-derived sSMCs, the presence of the *SRY* gene must be tested	Different clinical effects are expected in female and male carriers of X-chromosome-derived sSMCs, depending on the presence and functional status of *XIST.* Testing for the *SRY* gene and azoospermia factor (*AZF*) should be considered for patients with disorders of sex development who carry a Y-chromosome-derived sSMC.

## Data Availability

No new data were created or analyzed in this study. Data sharing is not applicable to this article.

## Abbreviations

The following abbreviations are used in this manuscript:

aCGH	Comparative Genome Hybridization
ACMG	American College of Medical Genetics and Genomics
ACP	Arm Chromosome Paint
AS	Angelman syndrome
AZF	Azoospermia factor
BFB	Breakage–Fusion–Bridge cycles
BP	Breakpoint hotspots
BPJs	Breakpoint junctions
cenM-FISH	Centromere-specific multicolor FISH
CES	Cat Eye Syndrome
CISS-hybridization	Chromosomal in situ suppression hybridization
DAPI	4′,6-diamidino-2-phenylindole
ECR	Euchromatic region
FISH	Fluorescence *in situ* hybridization
GTG-banding	G-banding by Trypsin and Giemsa
ITSs	Interstitial Telomeric Sequences
LCRs	Low-Copy Repeats
lrGS	long-read genome sequencing
MCB	MultiColor Banding
MMBIR	Microhomology-mediated break-induced replication
MMEJ	Microhomology-mediated end joining
NAHR	Non-allelic homologous recombination
NGS	Next-generation sequencing
NORs	Nucleolar Organizer Regions
OGM	Optical genome mapping
PKS	Pallister–Killian syndrome
PWS	Prader–Willi syndrome
rsSMC	Ring small supernumerary marker chromosome
SKY	Spectral karyotyping
SRY	Sex-determining region Y
sSMC	Small supernumerary marker chromosome
subcenM-FISH	Subcentromere-specific multicolor FISH
T2T	Telomere-to-telomere
TAD	Topological associated domain
UPD	Uniparental disomy
VCFS/DGS	Velo-Cardio-Facial/DiGeorge Syndrome
WCP	Whole-chromosome Paint
WGS	Whole-Genome Sequencing
XCl	X-chromosome inactivation
XIST	X-inactive specific transcript
